# Supplementary material for: SFX-01 is therapeutic against myeloproliferative disorders caused by activating mutations in Shp2
Source: EMBO Mol Med. 2025 Jul 10;17(8):2115–36. doi: 10.1038/s44321-025-00267-7 (PMC12340136; doi:10.1038/s44321-025-00267-7)
Supplement: Supplementary file 1 — Table EV1 [file 44321_2025_267_MOESM1_ESM.pdf]

Table EV1: Mass spectrometry data following LC-MS/MS of proteins adducted by SFN immunoprecipitated from cardiac tissue of WT mice following oral gavage with the electrophile.

Identified Proteins	Entry Name	kDa	P-Value	C1	C2	C3	C4	S1	S2	S3	S4	LOG2(SFN/CON)	LOG10(P-Value)
Myosin-binding protein C, cardiac-type	MYPC3_MOUSE	141	0.036	0	0	0	0	12	30	28	2	9.492	1.444
Isocitrate dehydrogenase [NADP], mitochondrial	IDHP_MOUSE	51	0.0001	0	0	0	0	18	13	19	18	9.409	4.000
Trifunctional enzyme subunit alpha, mitochondrial	ECHA_MOUSE	83	0.017	0	0	0	0	15	13	13	1	8.714	1.770
Pyruvate kinase PKM	KPYM_MOUSE	58	0.028	0	0	0	0	9	9	12	0	8.229	1.553
Aldehyde dehydrogenase, mitochondrial	ALDH2_MOUSE	57	0.067	0	0	0	0	1	4	8	16	8.180	1.174
Sarcoplasmic/endoplasmic reticulum calcium ATPase 2	AT2A2_MOUSE	115	0.012	0	0	0	0	6	11	10	2	8.180	1.921
Medium-chain specific acyl-CoA dehydrogenase, mitochondrial	ACADM_MOUSE	46	0.0027	0	0	0	0	8	3	5	8	7.907	2.569
Beta-enolase	ENOB_MOUSE	47	0.079	0	0	0	0	4	1	4	13	7.781	1.102
Pyruvate dehydrogenase E1 component subunit alpha, somatic form, mitochondrial	ODPA_MOUSE	43	0.00014	0	0	0	0	6	5	4	7	7.781	3.854
Electron transfer flavoprotein subunit alpha, mitochondrial	ETFA_MOUSE	35	0.00042	0	0	0	0	6	3	6	6	7.714	3.377
Isovaleryl-CoA dehydrogenase, mitochondrial	IVD_MOUSE	46	0.028	0	0	0	0	0	6	7	5	7.492	1.553
Fumarate hydratase, mitochondrial	FUMH_MOUSE	54	0.0013	0	0	0	0	2	5	5	5	7.409	2.886
Carnitine O-palmitoyltransferase 1, muscle isoform	CPT1B_MOUSE	88	0.029	0	0	0	0	4	5	6	0	7.229	1.538
Dihydrolipoyl dehydrogenase, mitochondrial	DLDH_MOUSE	54	0.0068	0	0	0	0	5	4	4	1	7.129	2.167
NADH-ubiquinone oxidoreductase 75 kDa subunit, mitochondrial	NDUS1_MOUSE	80	0.026	0	0	0	0	5	6	2	1	7.129	1.585

Pyruvate dehydrogenase E1 component subunit beta, mitochondrial	ODPB_MOUSE	39	0.16	0	0	0	0	1	0	9	3	7.022	0.796
BAG family molecular chaperone regulator 3	BAG3_MOUSE	62	0.13	0	0	0	0	2	8	2	0	6.907	0.886
Succinyl-CoA ligase [ADP-forming] subunit beta, mitochondrial	SUCB1_MOUSE	50	0.059	0	0	0	0	4	6	2	0	6.907	1.229
E3 ubiquitin-protein ligase NEDD4	NEDD4_MOUSE	103	0.048	0	0	0	0	2	5	4	0	6.781	1.319
Long-chain specific acyl-CoA dehydrogenase, mitochondrial	ACADL_MOUSE	48	0.08	0	0	0	0	4	1	0	5	6.644	1.097
Enoyl-CoA delta isomerase 1, mitochondrial	ECI1_MOUSE	32	0.0025	0	0	0	0	2	2	2	4	6.644	2.602
Glycogen phosphorylase, brain form	PYGB_MOUSE	97	0.053	0	0	0	0	5	2	3	0	6.644	1.276
Short-chain specific acyl-CoA dehydrogenase, mitochondrial	ACADS_MOUSE	45	0.039	0	0	0	0	0	2	3	4	6.492	1.409
Alpha-actinin-2	ACTN2_MOUSE	104	0.36	0	0	0	0	9	0	0	0	6.492	0.444
Calcium-binding mitochondrial carrier protein Aralar1	CMC1_MOUSE	75	0.17	0	0	0	0	0	3	6	0	6.492	0.770
Tubulin beta-4B chain	TBB4B_MOUSE	50	0.055	0	0	0	0	1	5	2	1	6.492	1.260
Mitochondrial inner membrane protein	IMMT_MOUSE	84	0.071	0	0	0	0	3	1	4	0	6.322	1.149
Tyrosine-protein phosphatase non-receptor type 11 (Shp2)	PTN11_MOUSE	68	0.03	0	0	0	0	3	3	2	0	6.322	1.523
Glycogen phosphorylase, muscle form	PYGM_MOUSE	97	0.071	0	0	0	0	3	1	4	0	6.322	1.149
Microtubule-associated protein 4	MAP4_MOUSE	117	0.14	0	0	0	0	3	4	0	0	6.129	0.854
Aspartate aminotransferase, mitochondrial	AATM_MOUSE	47	0.36	0	0	0	0	0	0	0	6	5.907	0.444
Annexin A2	ANXA2_MOUSE	39	0.36	0	0	0	0	0	0	0	6	5.907	0.444

NADH dehydrogenase [ubiquinone] 1 alpha subcomplex subunit 10, mitochondrial	NDUAA_MOUSE	41	0.059	0	0	0	0	1	2	0	3	5.907	1.229
Very long-chain specific acyl-CoA dehydrogenase, mitochondrial	ACADV_MOUSE	71	0.094	0	0	0	0	1	1	3	0	5.644	1.027
Mitochondrial 2-oxoglutarate/malate carrier protein	M2OM_MOUSE	34	0.36	0	0	0	0	0	0	0	5	5.644	0.444
182 kDa tankyrase-1-binding protein	TB182_MOUSE	182	0.36	0	0	0	0	0	4	0	0	5.322	0.444
2-oxoglutarate dehydrogenase, mitochondrial	ODO1_MOUSE	116	0.0005	0	0	1	0	10	9	10	5	5.087	3.301
Cardiomyopathy-associated protein 5	CMYA5_MOUSE	413	0.36	0	0	0	0	3	0	0	0	4.907	0.444
Isocitrate dehydrogenase [NAD] subunit alpha, mitochondrial	IDH3A_MOUSE	40	0.36	0	0	0	0	0	0	0	3	4.907	0.444
Prelamin-A/C	LMNA_MOUSE	74	0.36	0	0	0	0	0	3	0	0	4.907	0.444
Malate dehydrogenase, mitochondrial	MDHM_MOUSE	36	0.025	1	0	0	0	2	4	4	9	4.248	1.602
Cytochrome b-c1 complex subunit 1, mitochondrial	QCR1_MOUSE	53	0.001	0	0	3	0	12	19	12	10	4.143	3.000
Myosin-6	MYH6_MOUSE	224	0.024	0	5	8	1	15	93	94	45	4.141	1.620
Voltage-dependent anion-selective channel protein 2	VDAC2_MOUSE	32	0.0023	0	0	1	0	2	5	5	5	4.087	2.638
Myosin-7	MYH7_MOUSE	223	0.032	0	4	8	0	8	69	73	34	3.939	1.495
Trifunctional enzyme subunit beta, mitochondrial	ECHB_MOUSE	51	0.014	0	0	2	0	6	3	8	12	3.858	1.854
L-lactate dehydrogenase B chain	LDHB_MOUSE	37	0.023	0	0	2	0	8	6	2	12	3.807	1.638
Phosphoglycerate kinase 1	PGK1_MOUSE	45	0.031	0	0	1	0	2	3	5	1	3.459	1.509
LIM domain-binding protein 3	LDB3_MOUSE	76	0.01	0	0	0	2	7	7	5	2	3.392	2.000

ATP synthase subunit gamma, mitochondrial	ATPG_MOUSE	33	0.002	0	0	2	0	7	5	4	4	3.322	2.699
Heat shock cognate 71 kDa protein	HSP7C_MOUSE	71	0.093	2	0	0	0	7	10	3	0	3.322	1.032
3-ketoacyl-CoA thiolase, mitochondrial	THIM_MOUSE	42	0.0037	0	0	2	3	8	12	19	11	3.322	2.432
Glyceraldehyde-3-phosphate dehydrogenase	G3P_MOUSE	36	0.16	2	0	2	0	2	6	5	26	3.285	0.796
Acetyl-CoA acetyltransferase, mitochondrial	THIL_MOUSE	45	0.02	0	0	2	0	2	8	4	5	3.248	1.699
Cytochrome b-c1 complex subunit 2, mitochondrial	QCR2_MOUSE	48	0.0048	0	3	0	0	4	6	9	9	3.222	2.319
Succinate dehydrogenase [ubiquinone] flavoprotein subunit, mitochondrial	DHSA_MOUSE	73	0.047	0	0	3	0	10	8	9	0	3.170	1.328
Tubulin alpha-1C chain	TBA1C_MOUSE	50	0.066	0	0	1	0	2	3	4	0	3.170	1.180
Fructose-bisphosphate aldolase A	ALDOA_MOUSE	39	0.037	0	0	2	0	1	3	5	6	2.907	1.432
Myosin-8	MYH8_MOUSE	223	0.047	0	0	8	0	3	16	23	12	2.755	1.328
Myosin-4	MYH4_MOUSE	223	0.056	0	0	9	0	3	15	20	11	2.445	1.252
Perilipin-4	PLIN4_MOUSE	139	0.0039	0	0	1	4	8	8	6	5	2.433	2.409
ATP synthase subunit alpha, mitochondrial	ATPA_MOUSE	60	0.00025	4	8	25	6	48	60	64	54	2.394	3.602
ADP/ATP translocase 2	ADT2_MOUSE	33	0.016	0	0	5	0	6	4	7	8	2.322	1.796
2,4-dienoyl-CoA reductase, mitochondrial	DECR_MOUSE	36	0.13	0	0	2	0	5	0	2	3	2.322	0.886
Elongation factor Tu, mitochondrial	EFTU_MOUSE	50	0.048	0	0	4	0	2	6	5	4	2.087	1.319
Electron transfer flavoprotein-ubiquinone oxidoreductase, mitochondrial	ETFD_MOUSE	68	0.01	0	2	4	0	6	8	7	4	2.059	2.000
Aspartate aminotransferase, cytoplasmic	AATC_MOUSE	46	0.36	0	0	1	0	0	0	1	3	2.000	0.444

Phosphate carrier protein, mitochondrial	MPCP_MOUSE	40	0.028	2	0	2	2	5	6	10	3	2.000	1.553
ATP synthase subunit beta, mitochondrial	ATPB_MOUSE	56	0.024	0	1	18	7	13	24	30	36	1.986	1.620
Aconitate hydratase, mitochondrial	ACON_MOUSE	85	0.012	0	5	3	4	14	14	8	7	1.841	1.921
ADP/ATP translocase 1	ADT1_MOUSE	33	0.036	0	0	9	2	9	6	12	11	1.788	1.444
Actin, aortic smooth muscle	ACTA_MOUSE	42	0.11	1	0	4	22	12	15	19	25	1.395	0.959
Myosin light chain 3	MYL3_MOUSE	22	0.13	0	1	5	0	3	4	5	3	1.322	0.886
Stress-70 protein, mitochondrial	GRP75_MOUSE	73	0.27	0	0	4	1	3	5	4	0	1.263	0.569
Dihydrolipoyllysine-residue succinyltransferase component of 2-oxoglutarate dehydrogenase complex, mitochondrial	ODO2_MOUSE	49	0.24	3	12	26	24	15	25	23	46	0.746	0.620
Elongation factor 1-alpha 1	EF1A1_MOUSE	50	0.41	2	1	8	6	3	7	8	6	0.497	0.387
Pyruvate dehydrogenase protein X component, mitochondrial	ODPX_MOUSE	54	0.62	0	6	16	9	7	4	10	20	0.403	0.208
Actin, cytoplasmic 2	ACTG_MOUSE	42	0.79	0	0	0	28	5	11	6	14	0.363	0.102
Beta-actin-like protein 2	ACTBL_MOUSE	42	0.89	0	0	2	21	3	7	8	8	0.177	0.051
Dihydrolipoyllysine-residue acetyltransferase component of pyruvate dehydrogenase complex, mitochondrial	ODP2_MOUSE	68	0.4	21	80	122	110	46	51	61	87	-0.443	0.398
Multiple epidermal growth factor-like domains protein 8	MEGF8_MOUSE	297	0.56	1	3	0	5	0	2	2	2	-0.585	0.252
Kunitz-type protease inhibitor 2	SPIT2_MOUSE	28	0.67	0	2	0	0	0	0	0	1	-1.000	0.174

MAX gene-associated protein	MGAP_MOUSE	329	0.47	0	6	0	13	2	1	1	5	-1.078	0.328
Serine/threonine-protein kinase SIK3	SIK3_MOUSE	146	0.21	0	1	1	4	0	1	0	0	-2.585	0.678
Desmoplakin	DESP_MOUSE	333	0.18	6	0	0	10	0	0	1	0	-4.000	0.745
Protein diaphanous homolog 1	DIAP1_MOUSE	139	0.36	0	2	0	0	0	0	0	0	-4.322	0.444
Leucine-rich repeat-containing protein 16A	LR16A_MOUSE	152	0.36	0	0	0	2	0	0	0	0	-4.322	0.444
Ribosomal RNA processing protein 1 homolog B	RRP1B_MOUSE	81	0.36	0	0	2	0	0	0	0	0	-4.322	0.444
Histone H2B type 2-E	H2B2E_MOUSE	14	0.17	2	0	1	0	0	0	0	0	-4.907	0.770
Histone H3.3C	H3C_MOUSE	15	0.36	3	0	0	0	0	0	0	0	-4.907	0.444
Polyadenylate-binding protein 1	PABP1_MOUSE	71	0.36	3	0	0	0	0	0	0	0	-4.907	0.444
Desmoglein-4	DSG4_MOUSE	114	0.21	3	0	1	0	0	0	0	0	-5.322	0.678
Haptoglobin	HPT_MOUSE	39	0.36	0	0	0	4	0	0	0	0	-5.322	0.444
Proteoglycan 4	PRG4_MOUSE	116	0.36	0	0	0	5	0	0	0	0	-5.644	0.444
ATP-dependent RNA helicase DQX1	DQX1_MOUSE	79	0.36	0	0	0	6	0	0	0	0	-5.907	0.444
Plakophilin-1	PKP1_MOUSE	81	0.17	4	0	0	2	0	0	0	0	-5.907	0.770
Multidrug resistance protein 1A	MDR1A_MOUSE	141	0.36	0	0	0	7	0	0	0	0	-6.129	0.444
Histone H4	H4_MOUSE	11	0.17	6	0	3	0	0	0	0	0	-6.492	0.770
Histone H1.3	H13_MOUSE	22	0.16	7	0	4	0	0	0	0	0	-6.781	0.796
Myeloperoxidase	PERM_MOUSE	81	0.36	0	0	0	21	0	0	0	0	-7.714	0.444
Junction plakoglobin	PLAK_MOUSE	82	0.13	13	0	1	20	0	0	0	0	-8.409	0.886
