# Supplementary material for: SFX-01 is therapeutic against myeloproliferative disorders caused by activating mutations in Shp2
Source: EMBO Mol Med. 2025 Jul 10;17(8):2115–36. doi: 10.1038/s44321-025-00267-7 (PMC12340136; doi:10.1038/s44321-025-00267-7)
Supplement: Supplementary file 2 — Appendix [file 44321_2025_267_MOESM2_ESM.pdf]

Appendix

TABLE OF CONTENTS

1. Table of contents……………………………………………………………………………Page1

2. Appendix Figure S1………………………………………………………………………Page2

3. Appendix Figure S2………………………………………………………………………Page3

4. Appendix Figure S3………………………………………………………………………Page4

5. Appendix Figure S4………………………………………………………………………Page5

6. Appendix Figure S5………………………………………………………………………Page6

7. Appendix Figure S6………………………………………………………………………Page8

8. Appendix Figure S7………………………………………………………………………Page9

9. Appendix Figure S8………………………………………………………………………Page10

10. Appendix Figure S9……………………………………………………………………..Page11

11. Appendix Figure S10……………………………………………………………………Page12

12. Appendix Figure S11……………………………………………………………………Page13

13. Appendix Figure S12……………………………………………………………………Page14

14. Appendix Figure S13……………………………………………………………………Page15

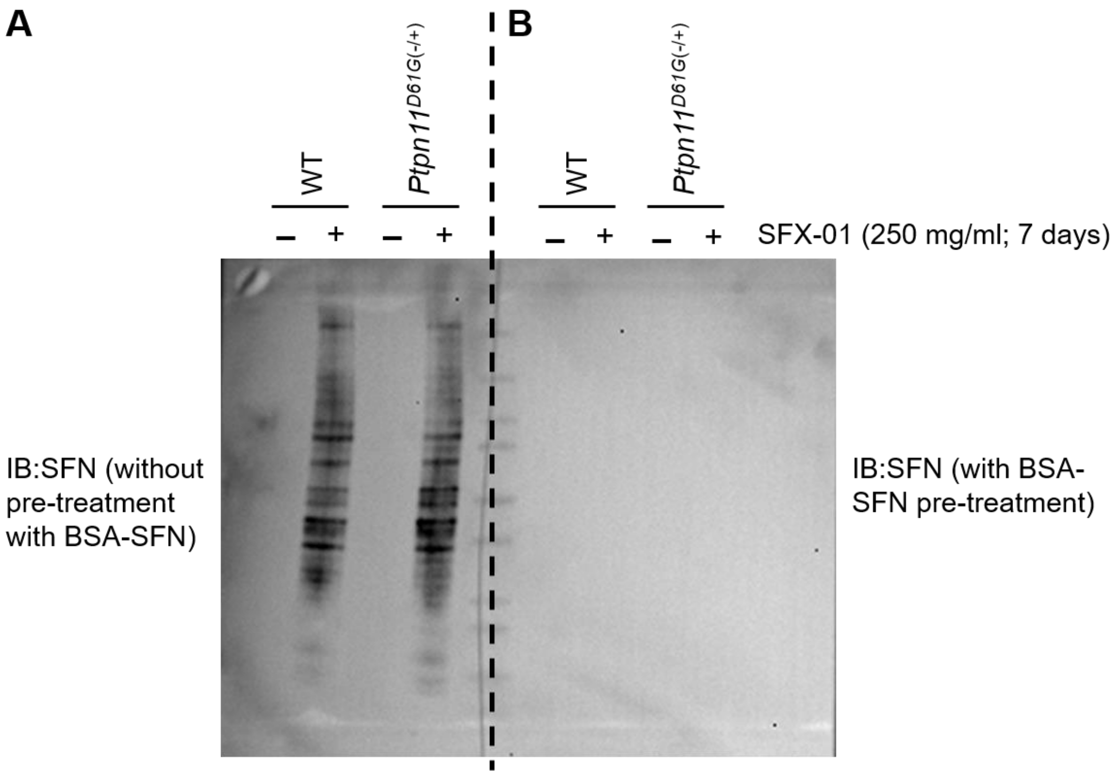

Appendix Figure S1. SFN-specific antibody

(A) SDS-PAGE immunoblot of cardiac tissue isolated from WT or *Ptpn11*$^{D61G(-/+)}$ mice after 7 days of SFX-01 (+) compared to untreated mice (-) incubated with cysteinyl-SFN specific rabbit polyclonal antibody. (B) Immunoblot as in (A) but incubated with cysteinyl-SFN specific rabbit polyclonal antibody pre-treated with SFN-labelled BSA (BSA-SFN) for 3 hours at 5°C.

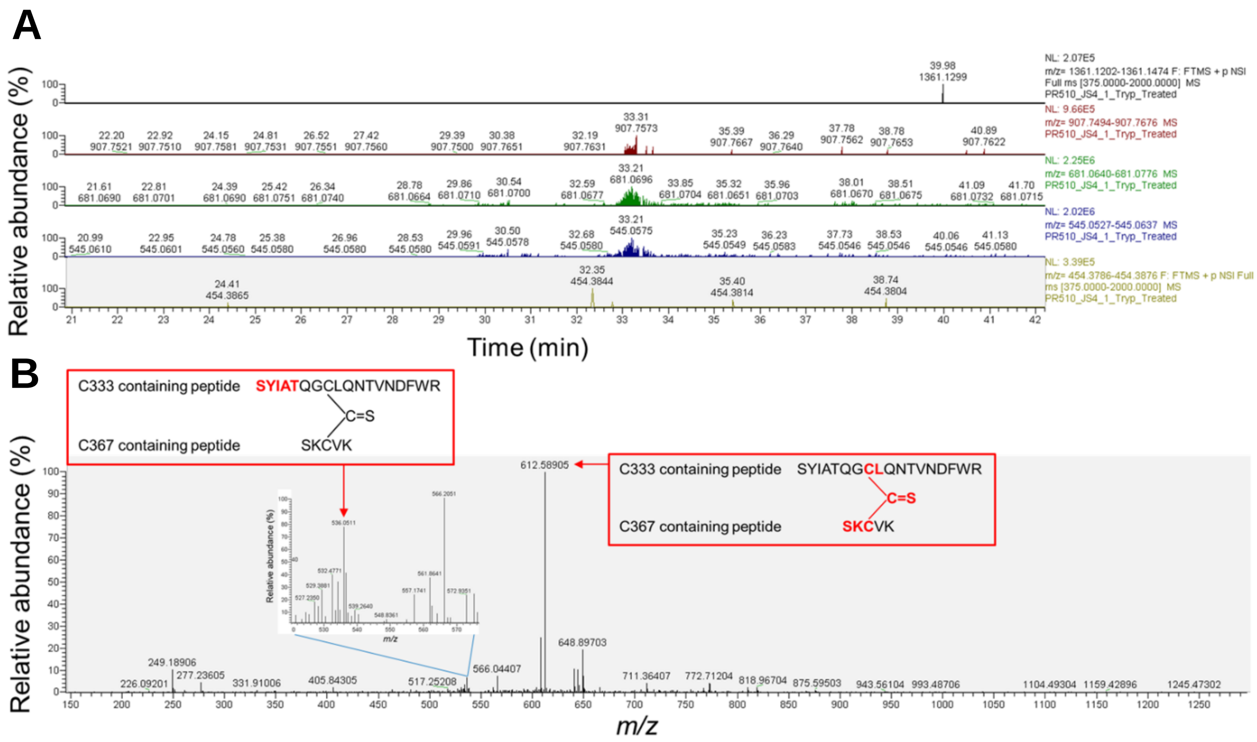

Appendix Figure S2. Dithiolethione formation in recombinant Shp2 following treatment with SFX-01

(A) LC-MS ion chromatograms of a tryptic digest of recombinant Shp2 incubated with equimolar SFX-01 for 6 hours, showing analyses of the sample performed at different charge states. These MS1 spectra were compared to the in silico calculated masses that would hypothetically be present if tryptic cysteine-containing peptides were conjoined and incorporated C=S from the isothiocyanate. This approach identified a product with an m/z of 907.75623 (when analyzing using a charge state of +3), 681.06964 (when analyzing using a charge state of +4) or 545.26074 (when analyzing using a charge state of +4), which is consistent with C333 and C367 being covalently linked in the way shown in Figure 2D to form a dithiolethione. (B) The putative dithiolethione-linked peptides were analyzed by MS2, fragmenting the parent ion into its constituent parts. If this peptide is truly a dithiolethione linking two tryptic peptides containing C333 and C367, the MS2 analysis should yield masses that can be definitively matched to fragments that would form from the parent ion. Two such MS2 ions (red arrows) were indeed identified, robustly corroborating a dithiolethione involving C333 and C367 of Shp2. The peptide sequence highlighted within the red boxes highlight the peptide sequences that match and underlie the MS2 ion spectra, which was collected with a charge of +5.

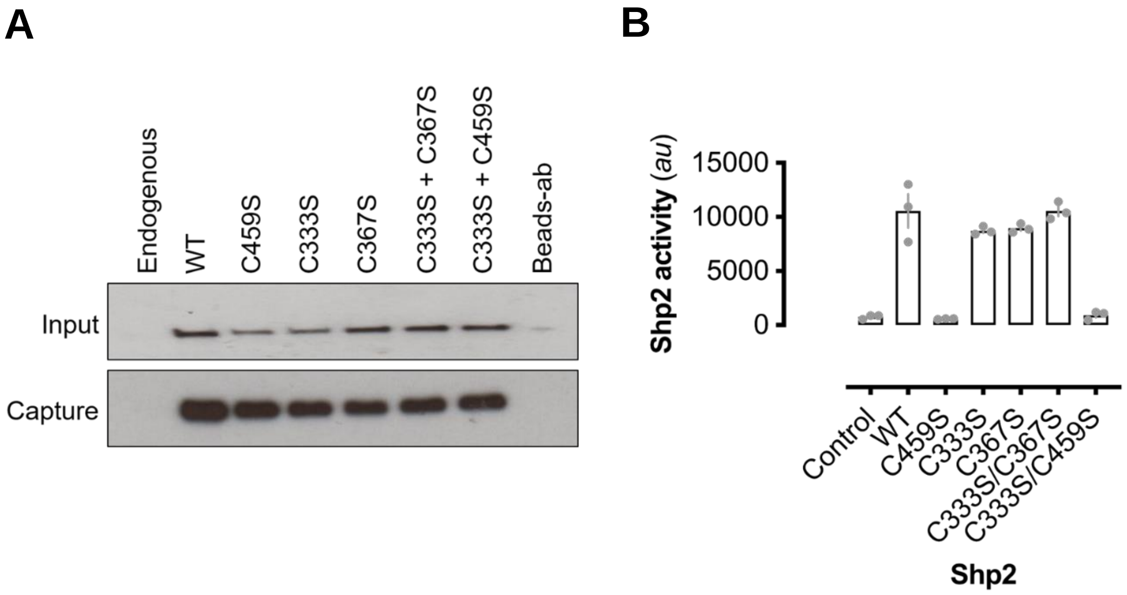

Appendix Figure S3. Mutant Shp2 activity

(A) SDS-PAGE immunoblot of input and immunoprecipitated Shp2 expressed in HEK293 cells used in downstream activity assay. (B) Graph representing immunoprecipitated Shp2 activity of WT and cysteine mutants. Bars represent mean activity (± SEM; n=3).

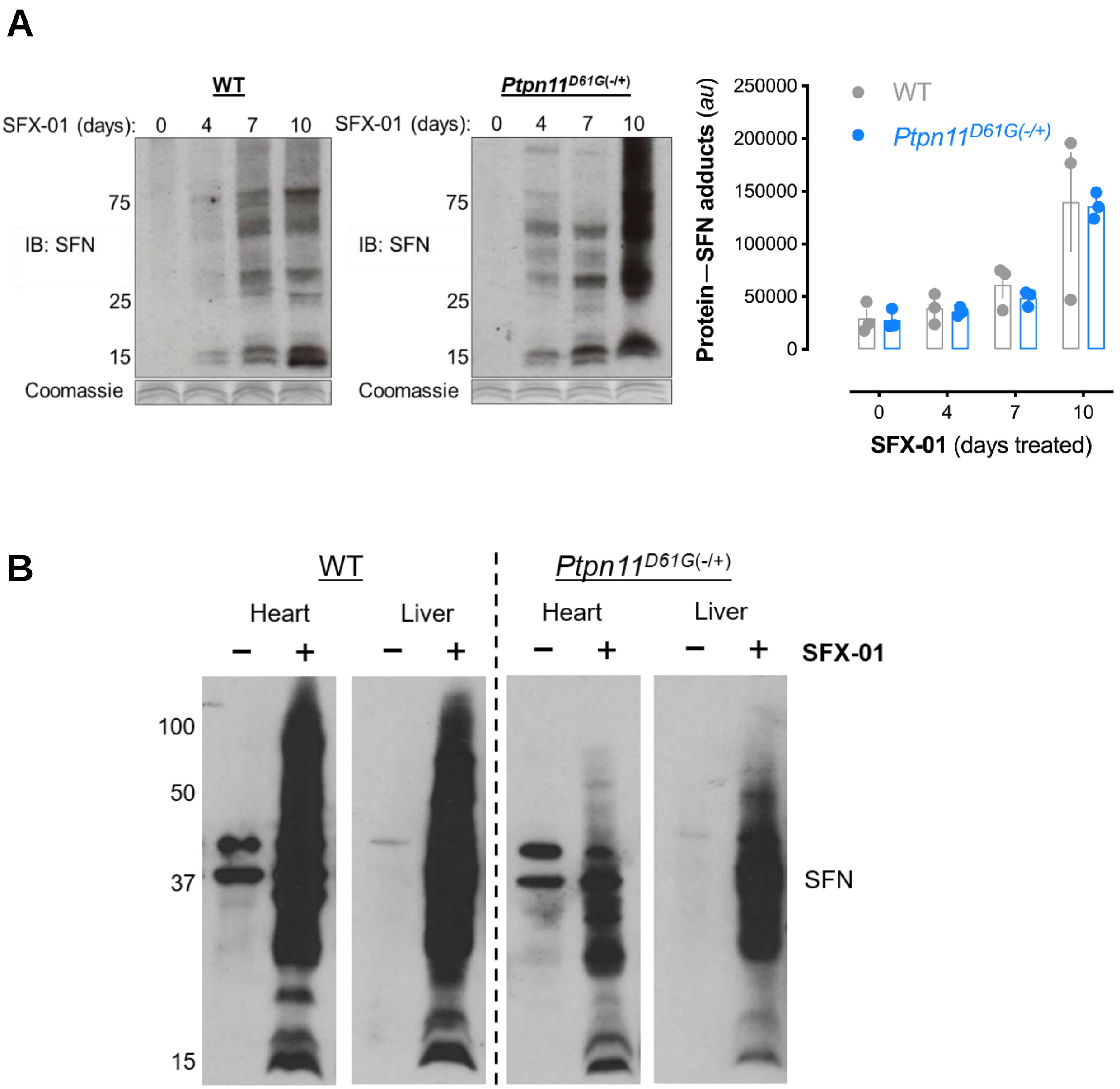

Appendix Figure S4. SFX-01 administration results in SFN-labelled protein

(A) Representative immunoblots and densitometric bar graph of SFN-labelled cardiac proteins isolated from WT or *Ptpn11*$^{D61G(-/+)}$ mice treated with SFX-01 for 0-10 days. (B) Immunoblots of SFN-labelled cardiac and liver proteins isolated from WT or *Ptpn11*$^{D61G(-/+)}$ mice treated with vehicle or SFX-01 for 10 days.

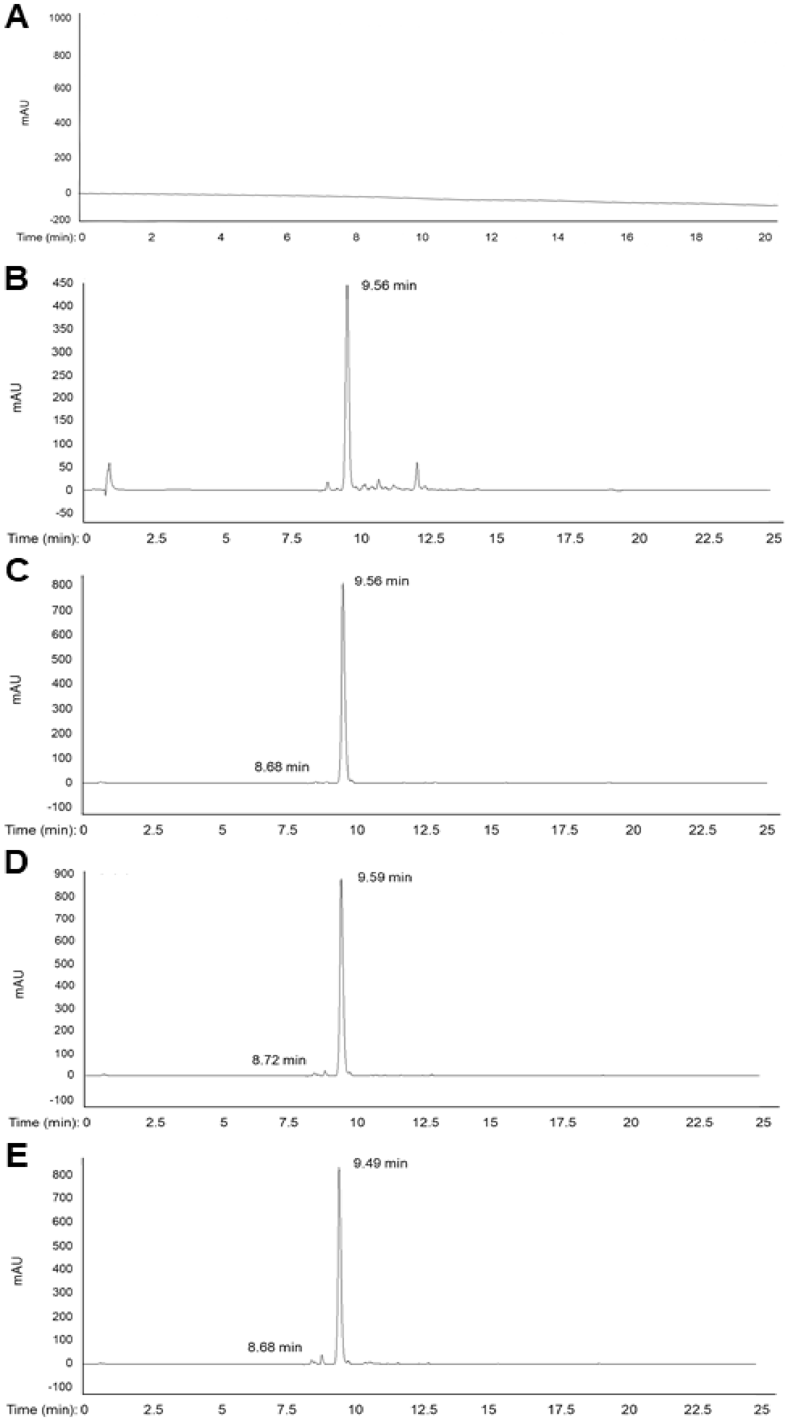

Appendix Figure S5. Stability of SFX-01 in water

HPLC chromatograms of (A) water only negative control, (B) 0.385 mg/ml L-SFN in water, serving as a positive control. (C-E) 2.5 mg/ml SFX-01 dissolved in water, stored at room temperature and analyzed at time points of 0, 4 and 6 days respectively. A small peak with a retention time of ~8.70

mins, which likely represents a minor amount of degradation, became apparent after 4 days. mAU represents absorbance using a UV detector set at a wavelength of 205 nm.

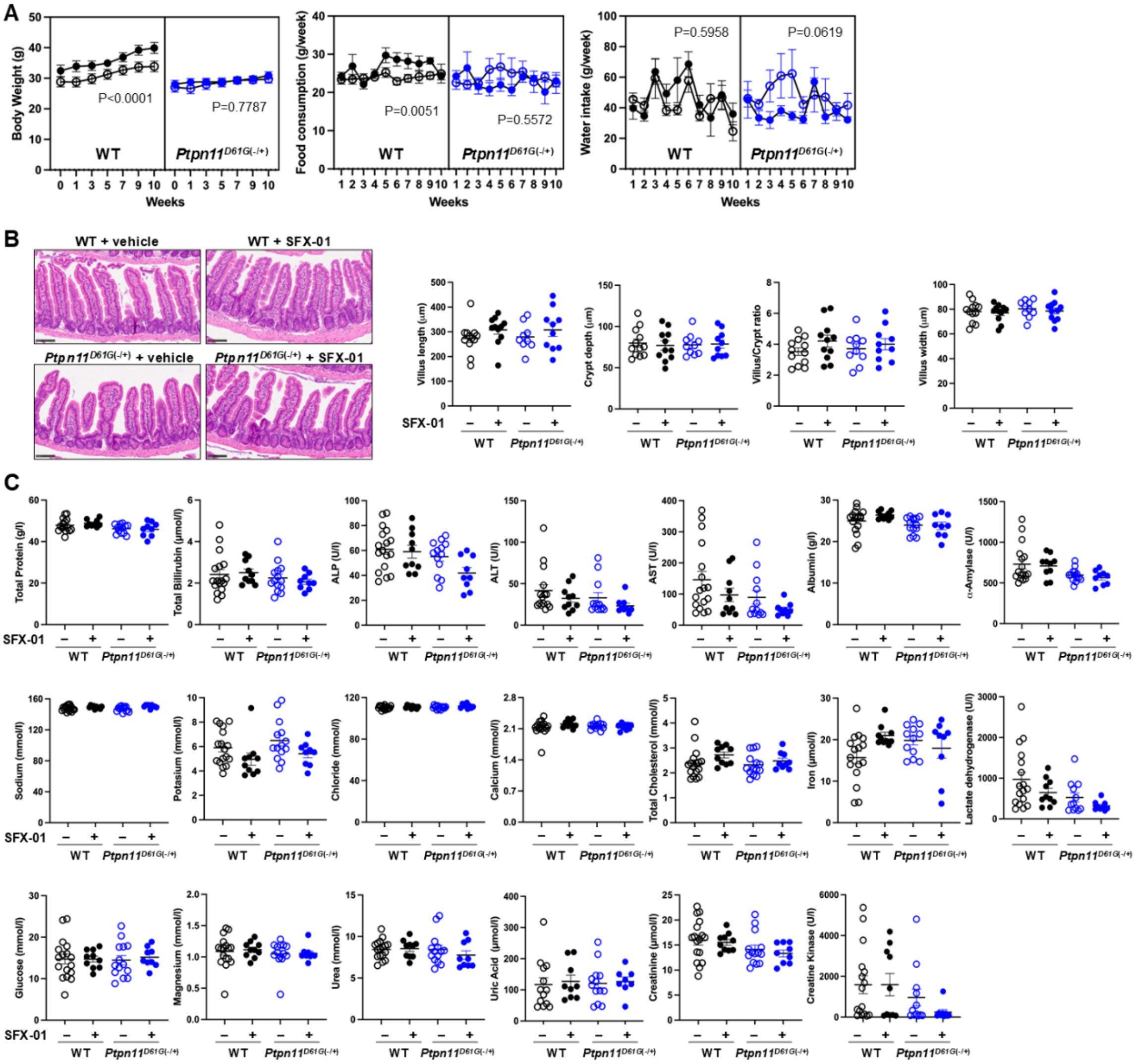

Appendix Figure S6. *In vivo* SFX-01 toxicity analysis

WT or *Ptpn11*$^{D61G(-/+)}$ mice treated with 2.5 mg/ml SFX-01 in their drinking water for 10 weeks. (A) Body weight, food consumption and water intake of mice over time. WT + vehicle is black open circle, WT + SFX-01 is black circle, *Ptpn11*$^{D61G(-/+)}$ + vehicle is blue open circle and *Ptpn11*$^{D61G(-/+)}$ + SFX-01 is blue circle. Data shown are mean ± SEM and P-values calculated by two-way ANOVA with Tukey's multiple comparison test. (B) H&E staining of small intestine after treatment and the length and width of villus and length of crypts were measured. Scale bars show 250 µm (C) Impact of SFX-01 treatments on liver, cardiac and renal function biomarkers in mice: alkaline phosphatase (ALP), alanine aminotransferase (ALT), aspartate aminotransferase (AST), Data are presented as means (±SEM n=9-17 per group).

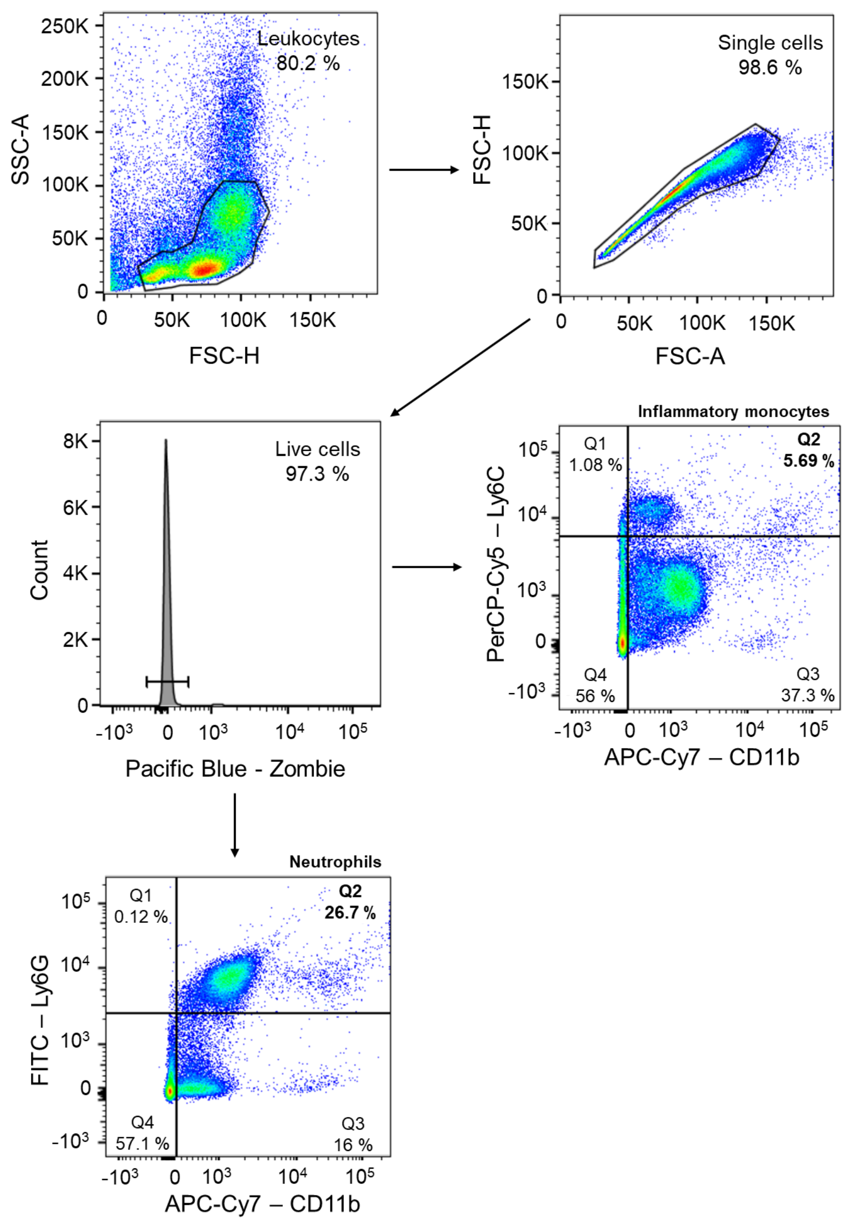

Appendix Figure S7. Gating strategy for *in vivo* myeloid cell measurements

Representative FLOW-JO plots and histograms showing gates used to calculate percentages of each cell population within the blood from a 22 week-old *Ptpn11*$^{D61G(-/+)}$ mouse. Leukocytes (SSC-A vs FSC-H), single cells (FSC-H vs FSC-A), live cells (Cell count vs Pacific Blue), inflammatory monocytes (CD11b^{+}Ly6C^{Hi}) and neutrophils (CD11b^{+}Ly6G^{Hi}). Values represent percentage of the previous gate. For example, 5.69% of live, single-cell leukocytes are inflammatory monocytes.

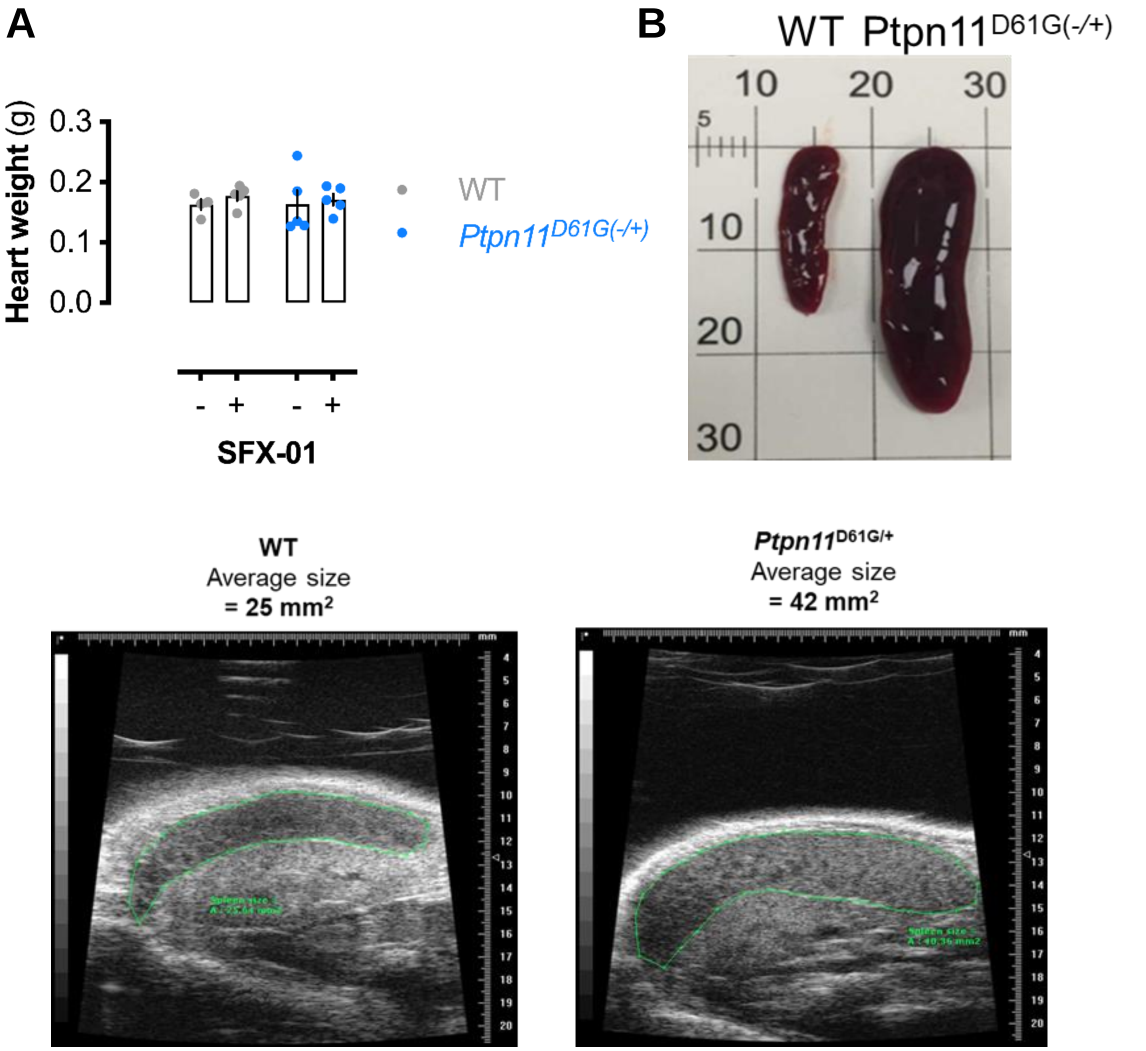

Appendix Figure S8. Splenomegaly in Shp2 mutant mice

(A) Graph showing heart weight of WT or *Ptpn11*$^{D61G(-/+)}$ mice treated with or without SFX-01. Bars represent mean weight (± SEM; n=5). (B) Representative spleen from WT or *Ptpn11*$^{D61G(-/+)}$ mice. (C) Ultrasound image of spleens in vivo of WT or *Ptpn11*$^{D61G(-/+)}$ mice.

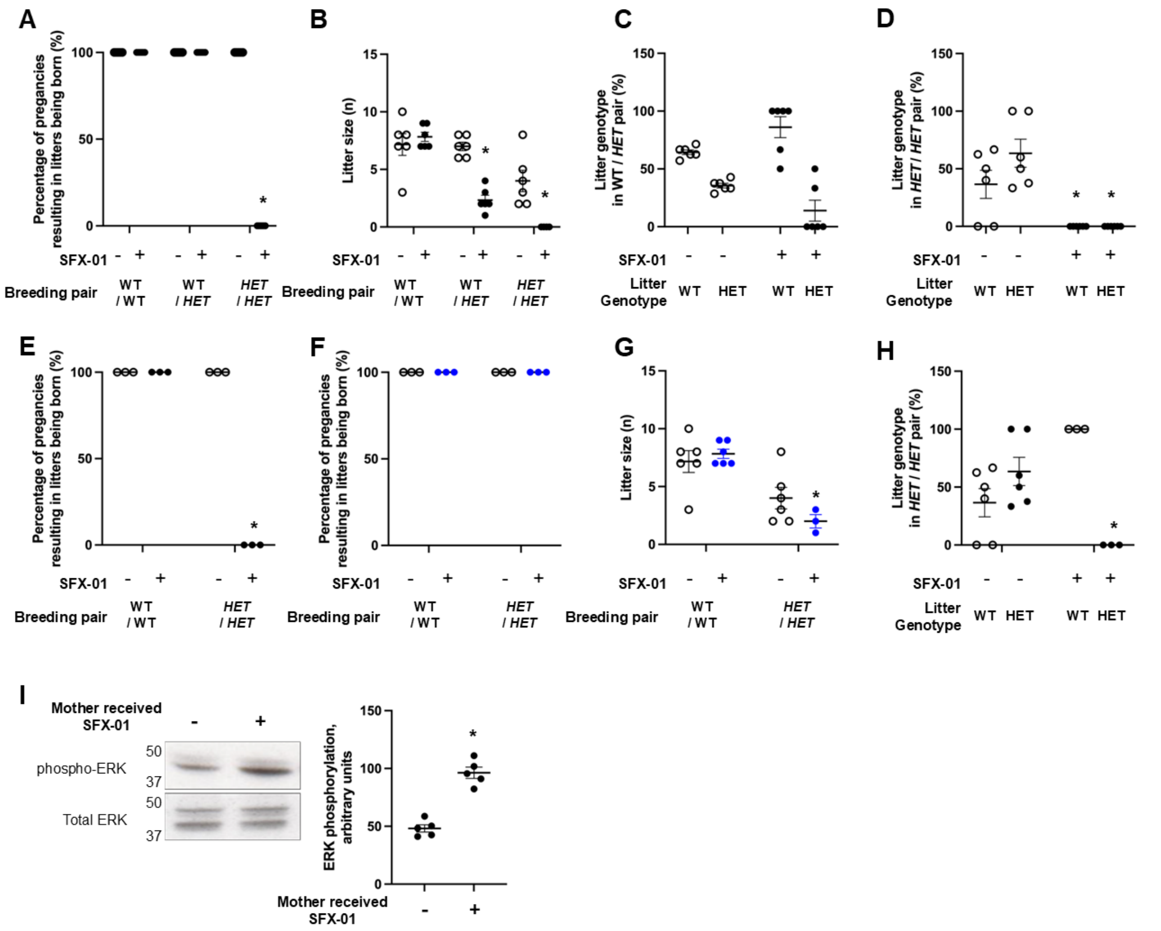

Appendix Figure S9. Fetal treatment with SFX-01 is embryonic lethal in pregnancies carrying NS fetuses.

(A) Treatment of heterozygous *Ptpn11*$^{D61G(-/+)}$ / *Ptpn11*$^{D61G(-/+)}$ breeding pairs with 2.5 mg/ml SFX-01 in their drinking water before and during pregnancy resulted in no litters being born. (B-D) Treatment of WT / heterozygous *Ptpn11*$^{D61G(-/+)}$ breeding pairs with 2.5 mg/ml SFX-01 in their drinking water before and during pregnancy significantly reduced litter size with a trend for a lower percentage of heterozygous offspring. (n=6, *P<0.05 versus untreated control). (E) Treatment of heterozygous *Ptpn11*$^{D61G(-/+)}$ / *Ptpn11*$^{D61G(-/+)}$ breeding pairs with 2.5 mg/ml SFX-01 in their drinking water 11 days post-conception resulted in no litters being born. (n=3, *P<0.05 versus untreated control). (F) Percentage of pregnancies resulting in the birth of litters was not altered following treatment of WT / WT or heterozygous *Ptpn11*$^{D61G(-/+)}$ / *Ptpn11*$^{D61G(-/+)}$ breeding pairs with 0.8 mg/ml SFX-01 before and during pregnancy. (G-H) Treatment of heterozygous *Ptpn11*$^{D61G(-/+)}$ / *Ptpn11*$^{D61G(-/+)}$ breeding pairs with 0.8 mg/ml SFX-01 significantly reduced litter size with only WT offspring born. (n=6, *P<0.05 versus untreated control). (I) An immunoblot showing increased ERK phosphorylation in WT neonates from WT / WT breeding pairs who had received 0.8 mg/ml SFX-01 in their drinking water before and during pregnancy. (n=5, *P<0.05 versus untreated control). HET means heterozygous *Ptpn11*$^{D61G(-/+)}$.

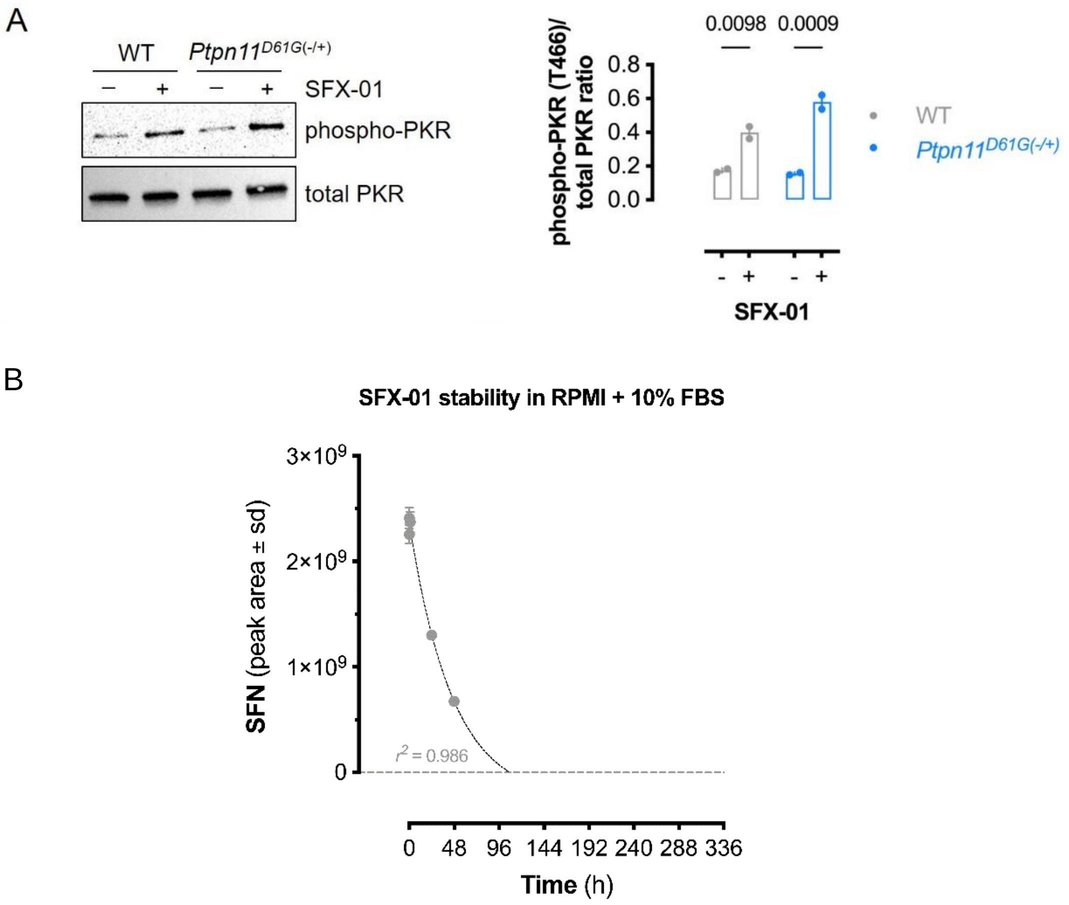

Appendix Figure S10. SFX-01 induces cell cycle arrest

(A) Immunoblot of relative PKR phosphorylation in leukocytes isolated from WT or *Ptpn11$^{D61G(-/+)}$* mice treated with or without SFX-01 for 7 days. Graph represents mean relative PKR phosphorylation levels (± sd; n=2). (B) Graph depicting free SFN from SFX-01 after incubation at 37°C in RPMI media containing 10% FBS for the times indicated. SFN was measured by LC-MS after extracting media with 80% methanol. Data shown are mean SFN chromatograph peak areas (±sd; n=4) and data was fitted to a first-order decay curve using Prism 9.

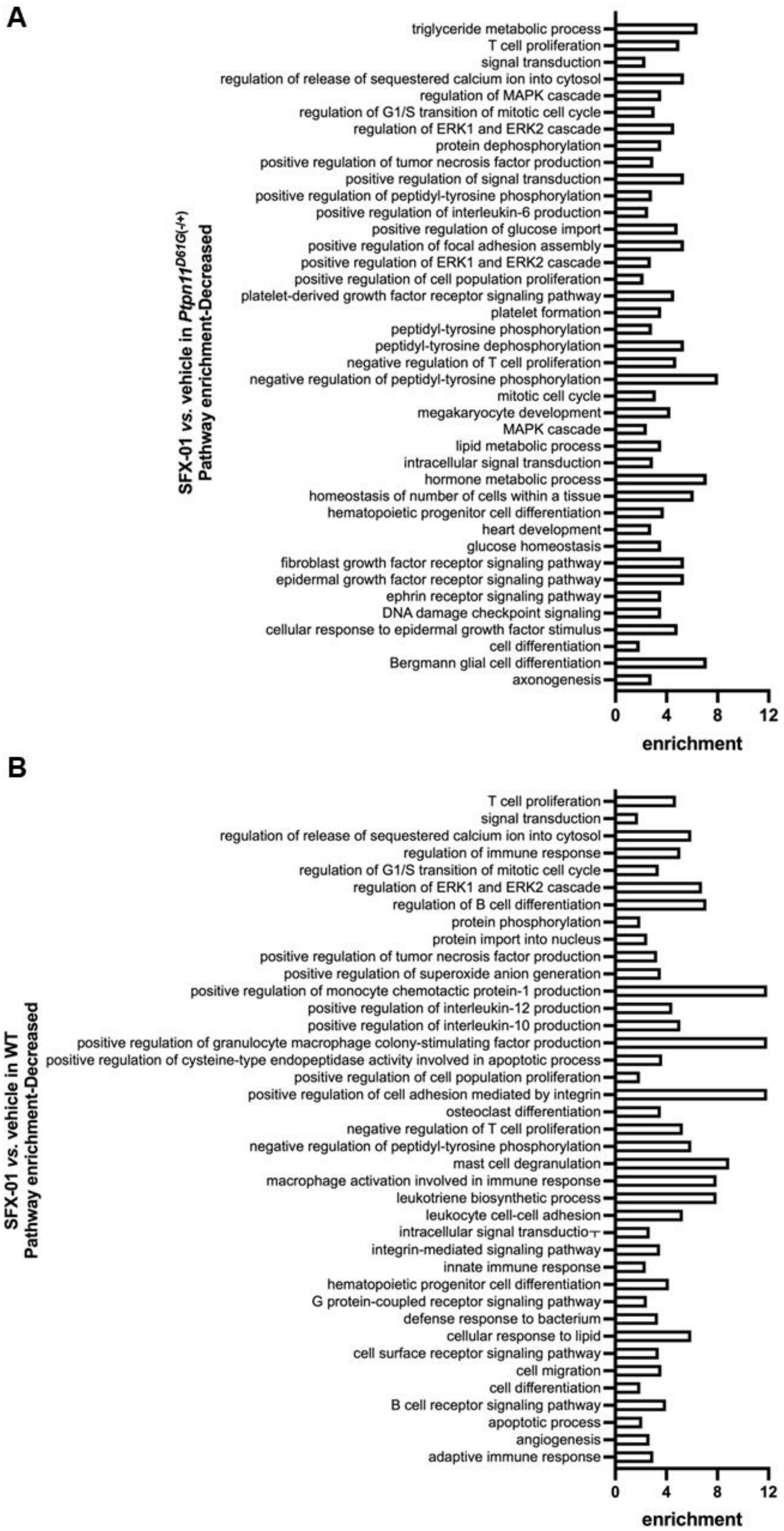

Appendix Figure S11. Gene ontology analysis of significantly downregulated pathways mediated by Shp2 following SFX-01 treatment

(A) Downregulated pathways in CD11b+ cells from *Ptpn11*$^{D61G(-/+)}$ mice treated with SFX-01 compared to the vehicle. (B) Downregulated pathways in CD11b+ cells from WT mice treated with SFX-01 compared to the vehicle.

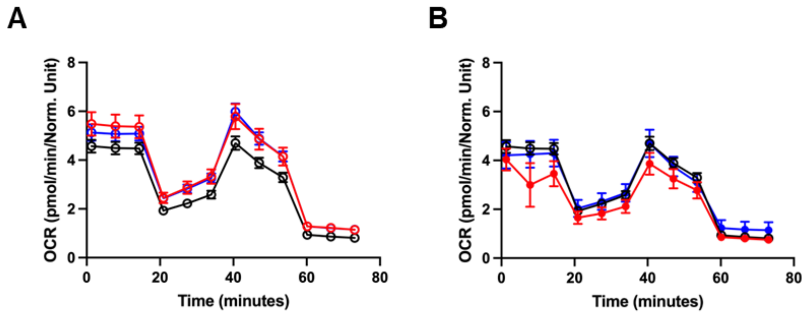

Appendix Figure S12. SFX-01 and SHP099 did not alter mitochondrial function

Treatment with 3 µM or 10 µM SFX-01(A) or SHP099 (B) did not significantly alter mitochondrial function as indexed by oxygen consumption rate (OCR). The vehicle control is represented by black open circles, 3 µM SFX-01 by red open circles, 10 µM SFX-01 by blue open circles, 3 µM SHP099 by solid red circles, and 10 µM SHP099 by solid blue circles.

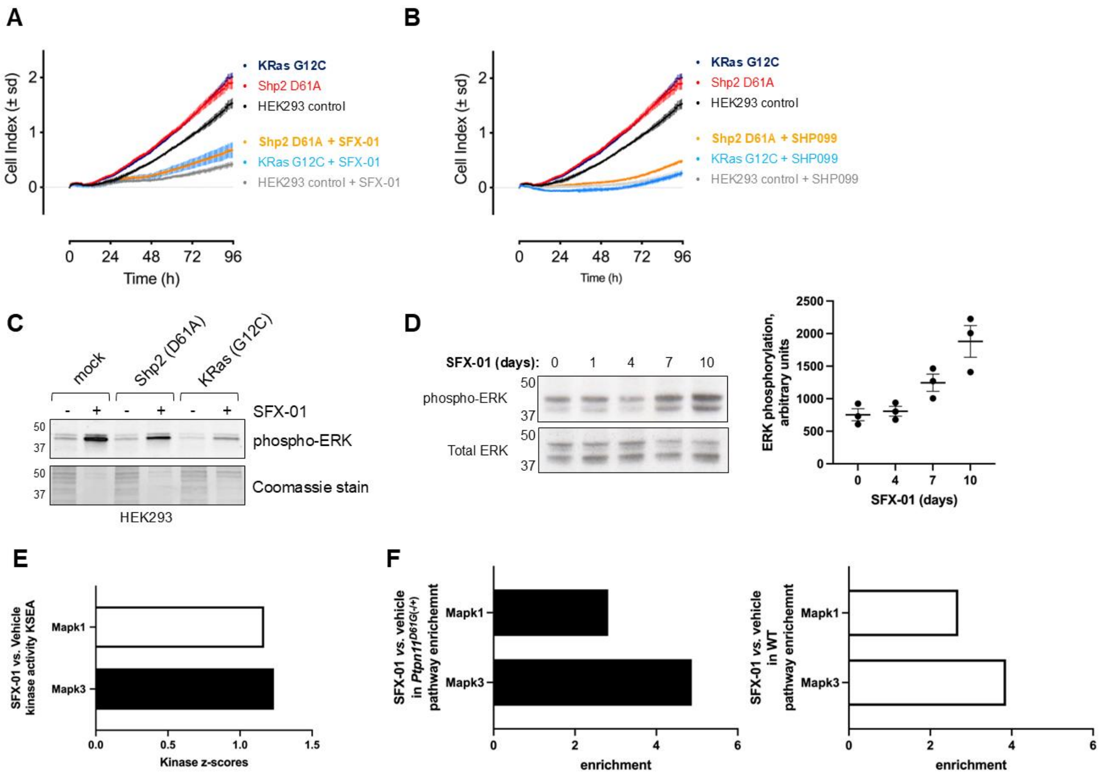

Appendix Figure S13. SFX-01 and SHP099 have similar effects on cellular proliferation

(A-B) The proliferation of HEK293 cell expressing mutant Shp2 or KRas treated without or with 12.5 µM SFX-01 or Shp099 for 96 hours. Data represent mean cell index values (± sd; n=2-4). (C) Immunoblot of phospho-ERK expression in HEK293 cells expressing either mutant Shp2 or KRas and compared to untransfected controls cells with or without SFX-01 (12.5 µM) for 96 hours. Coomassie stained immunoblot shows relative protein loaded per lane. (D) An Immunoblot showing increased ERK phosphorylation in cardiac tissue of WT mice following treatment with 2.5 mg/ml SFX-01 for 10 days in their drinking water. (n=5, *P<0.05 versus water only control). (E) Phosphoproteomics kinase-substrate enrichment analysis (KSEA) showed a significant increase in Mapk1 phosphorylation in SFX-01-treated CD11b+ WT bone marrow cells (represented by the open bar) and an elevation in Mapk3 phosphorylation in SFX-01-treated CD11b+ *Ptpn11*$^{D61G(-/+)}$ bone marrow cells (represented by the black bar). (F) KSEA pathway enrichment analysis indicated significant enrichment of the MAPK signaling pathway in both WT and *Ptpn11*$^{D61G(-/+)}$ CD11b+ bone marrow cells following SFX-01 treatment.
