# Supplementary figures and images for: SFX-01 is therapeutic against myeloproliferative disorders caused by activating mutations in Shp2
Source: EMBO Mol Med. 2025 Jul 10;17(8):2115–36. doi: 10.1038/s44321-025-00267-7 (PMC12340136; doi:10.1038/s44321-025-00267-7)

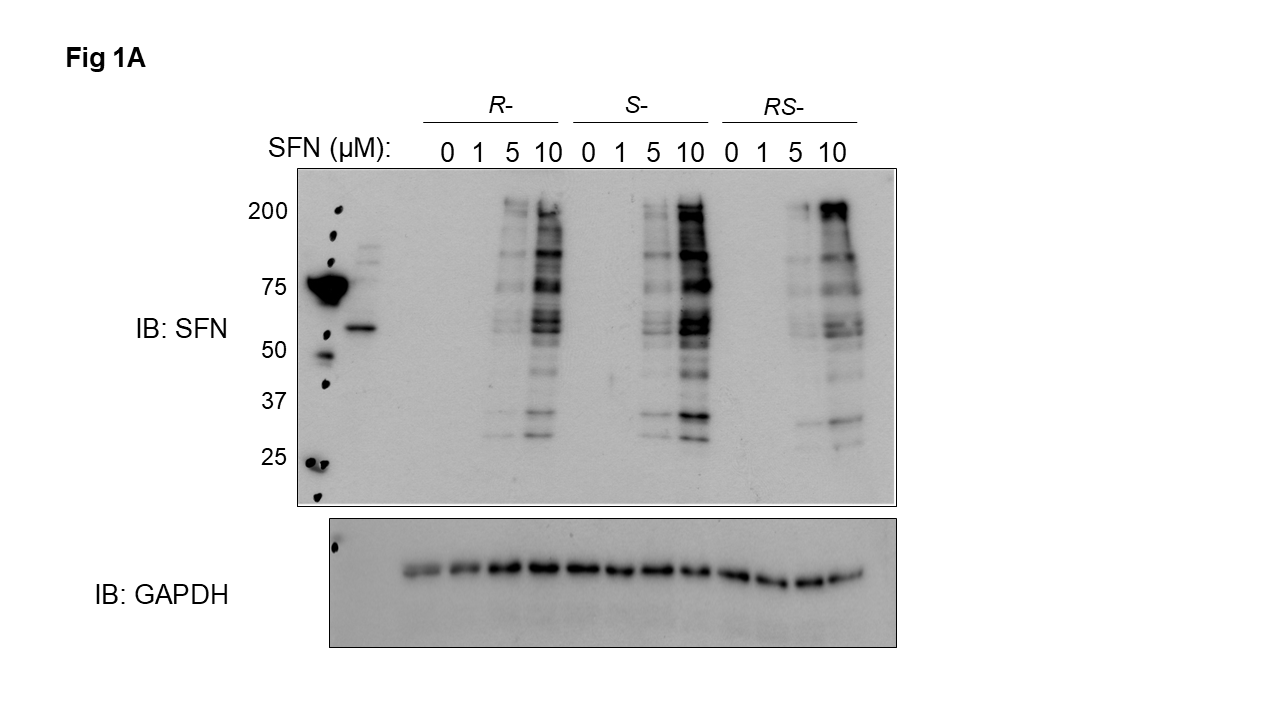

Supplement: Supplementary file 4 — Source data Fig. 1 [file 44321_2025_267_MOESM4_ESM.zip › Fig1/Fig1A.tif]

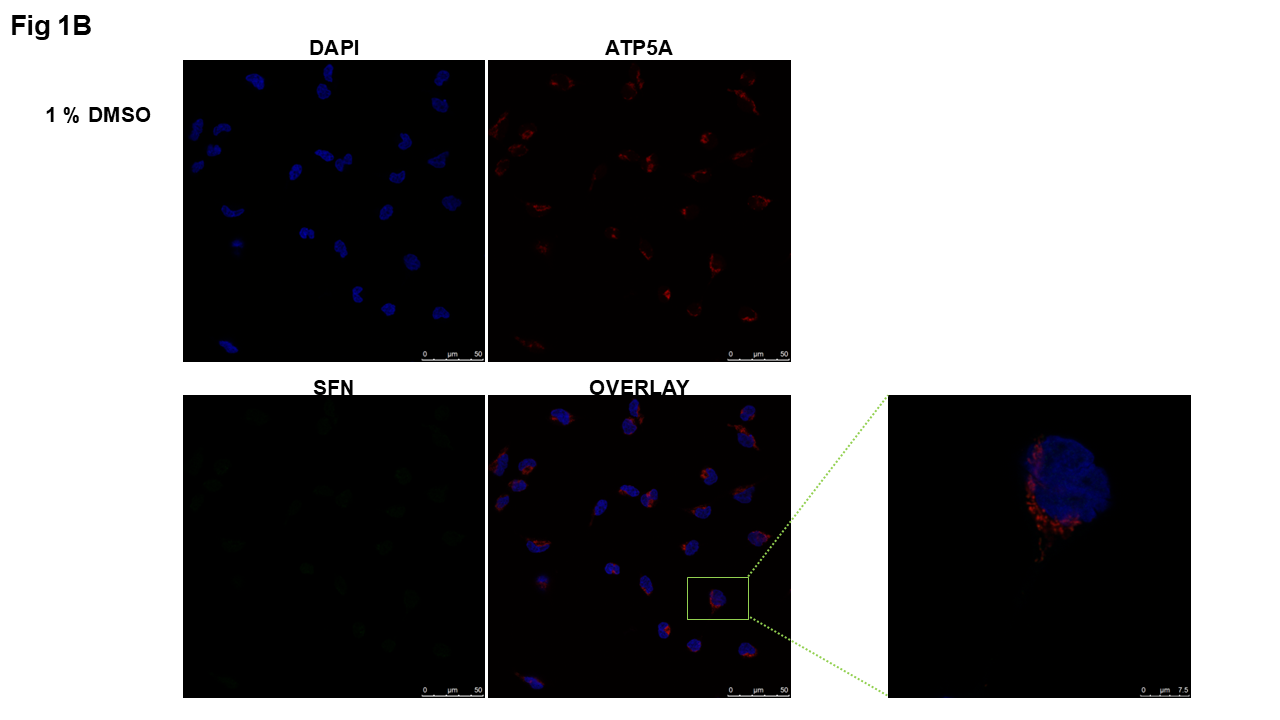

Supplement: Supplementary file 4 — Source data Fig. 1 [file 44321_2025_267_MOESM4_ESM.zip › Fig1/Fig1B-1 % DMSO.TIF]

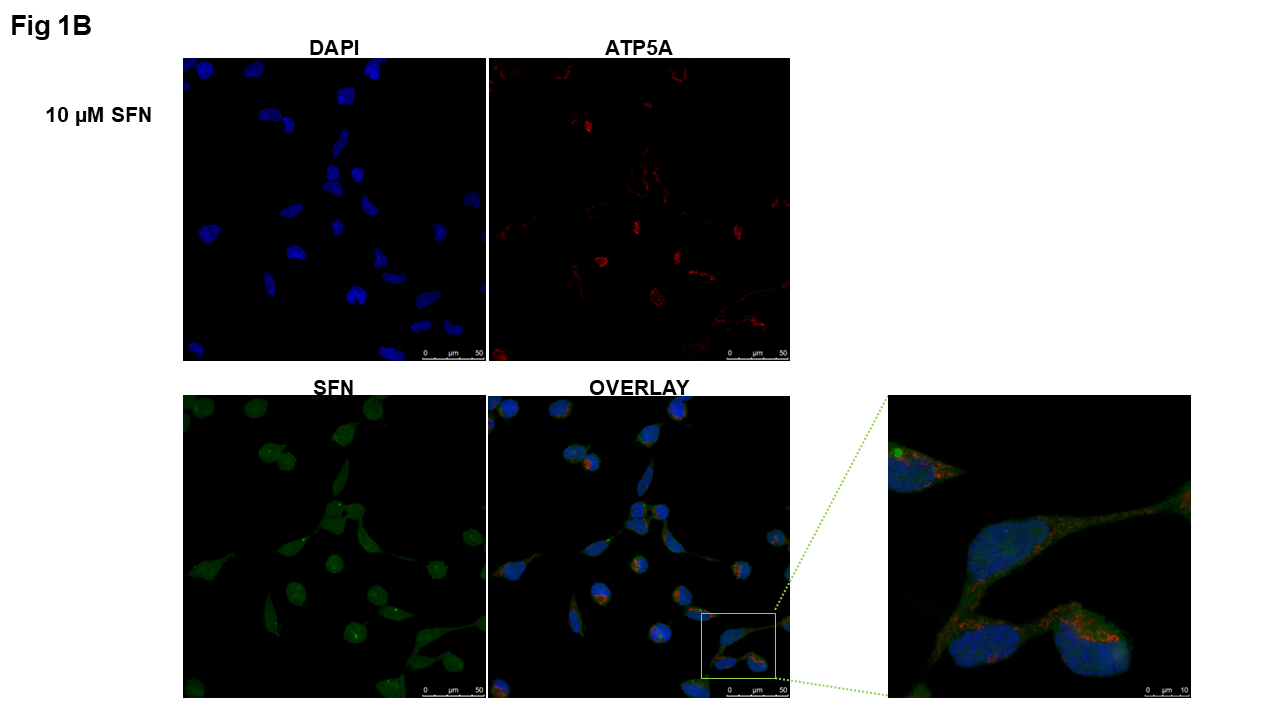

Supplement: Supplementary file 4 — Source data Fig. 1 [file 44321_2025_267_MOESM4_ESM.zip › Fig1/Fig1B-10 uM SFN.TIF]

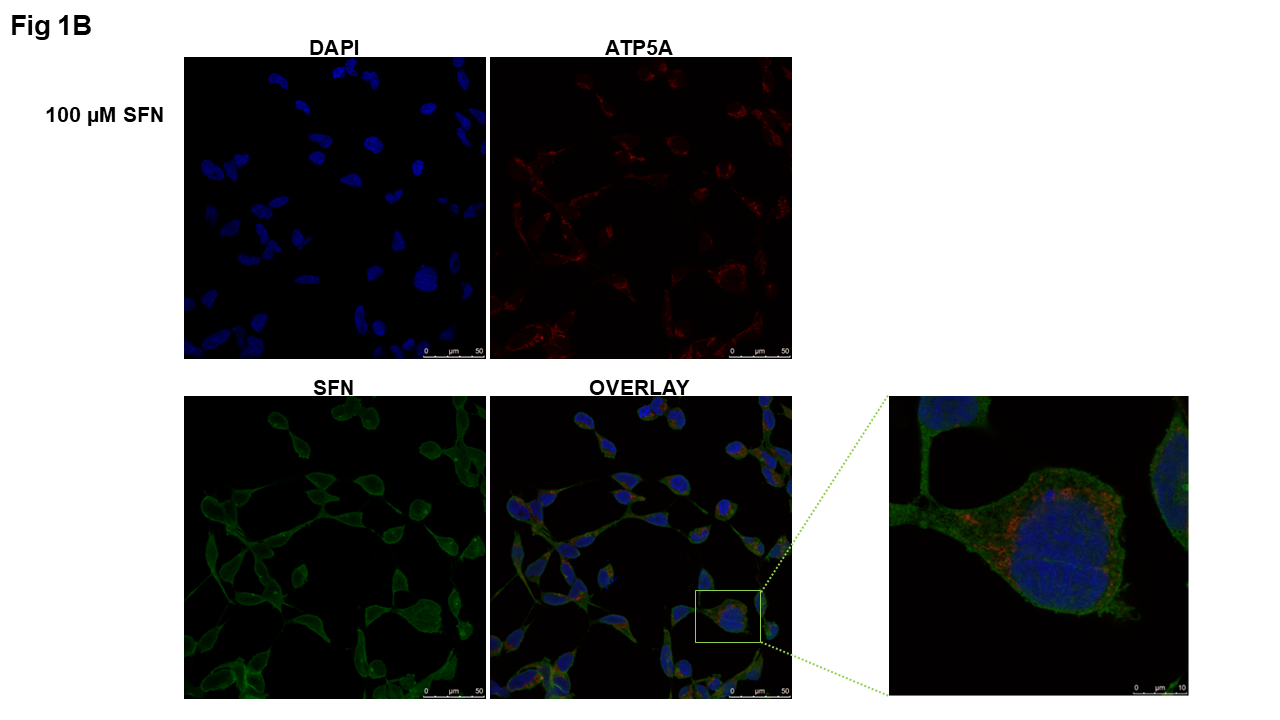

Supplement: Supplementary file 4 — Source data Fig. 1 [file 44321_2025_267_MOESM4_ESM.zip › Fig1/Fig1B-100 uM SFN.TIF]

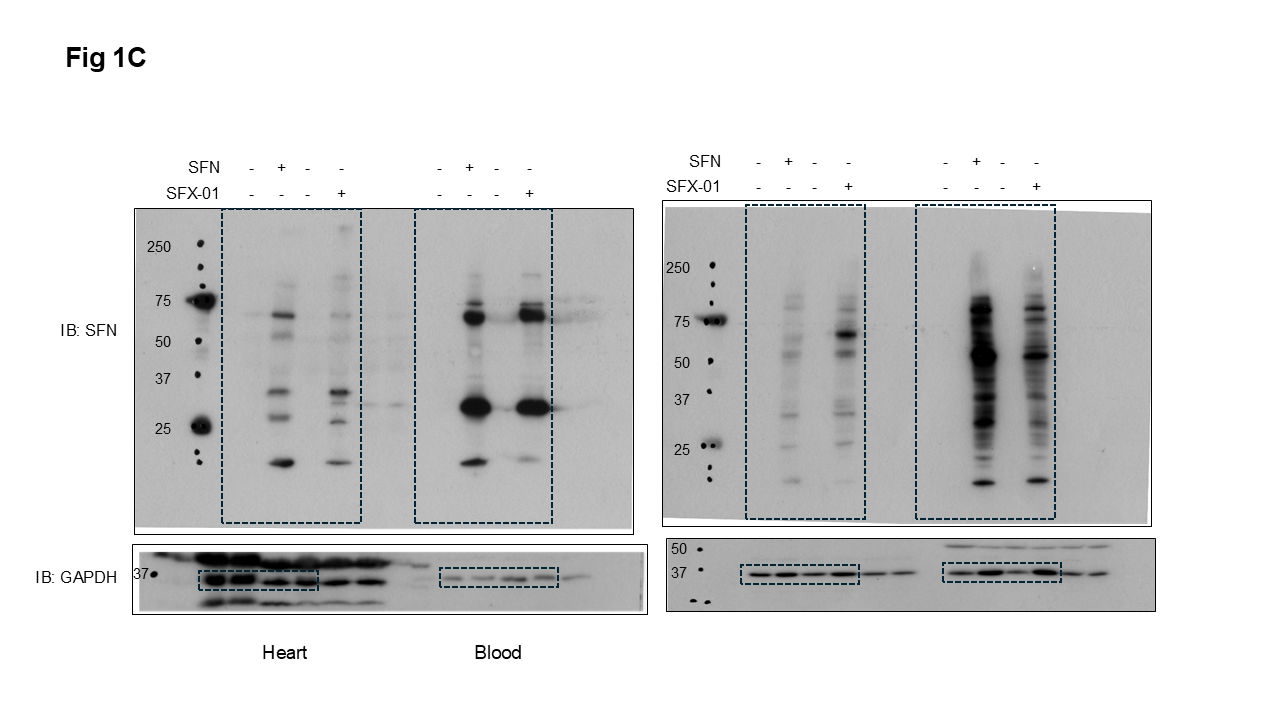

Supplement: Supplementary file 4 — Source data Fig. 1 [file 44321_2025_267_MOESM4_ESM.zip › Fig1/Fig1C.tif]

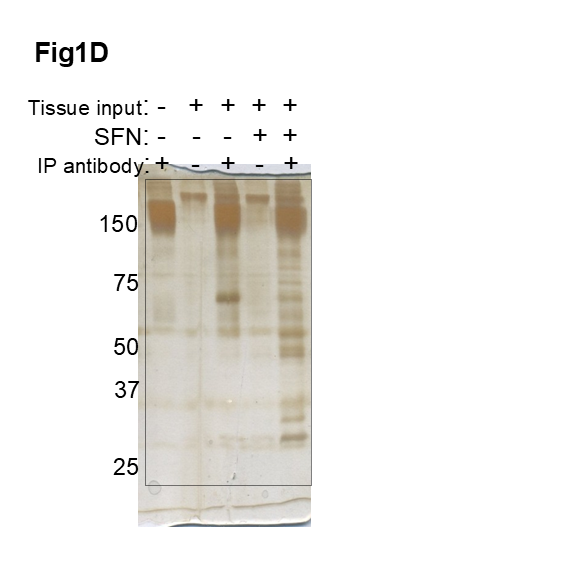

Supplement: Supplementary file 4 — Source data Fig. 1 [file 44321_2025_267_MOESM4_ESM.zip › Fig1/Fig1D.tif]

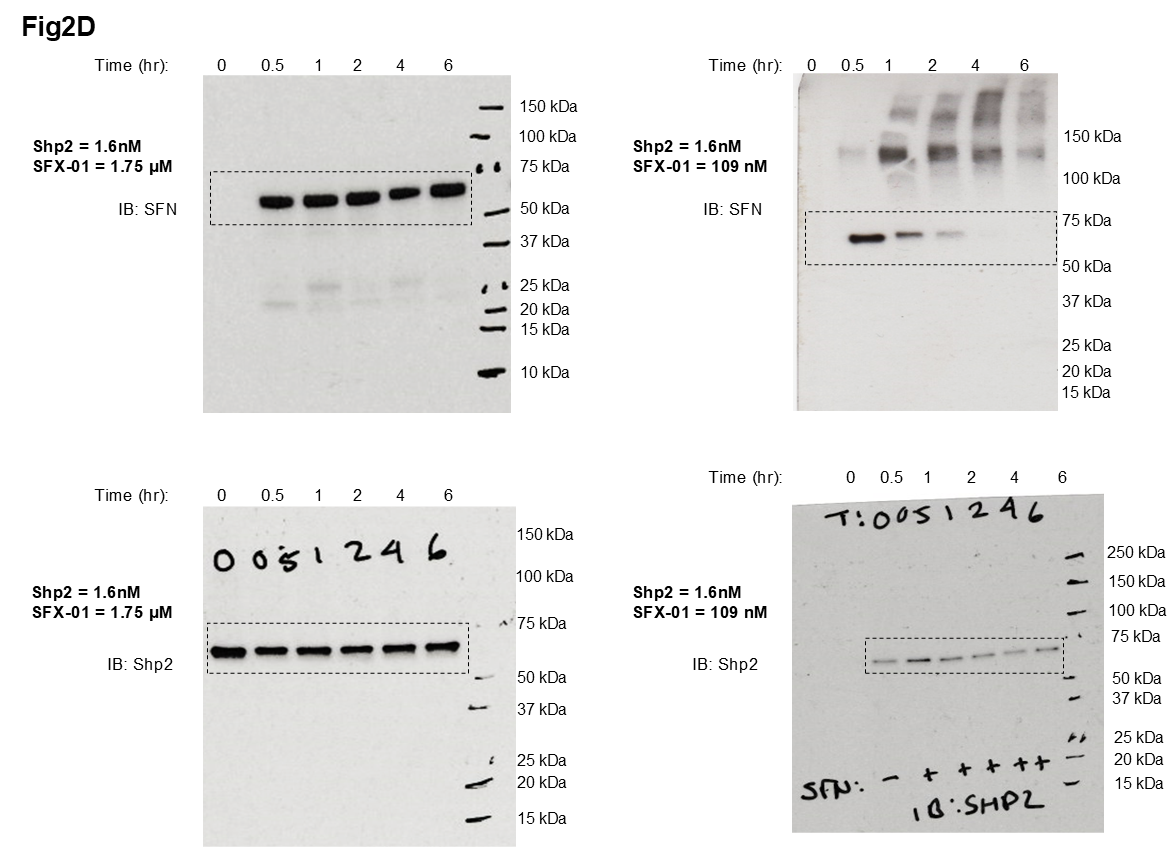

Supplement: Supplementary file 5 — Source data Fig. 2 [file 44321_2025_267_MOESM5_ESM.zip › Fig2/Fig2D.tif]

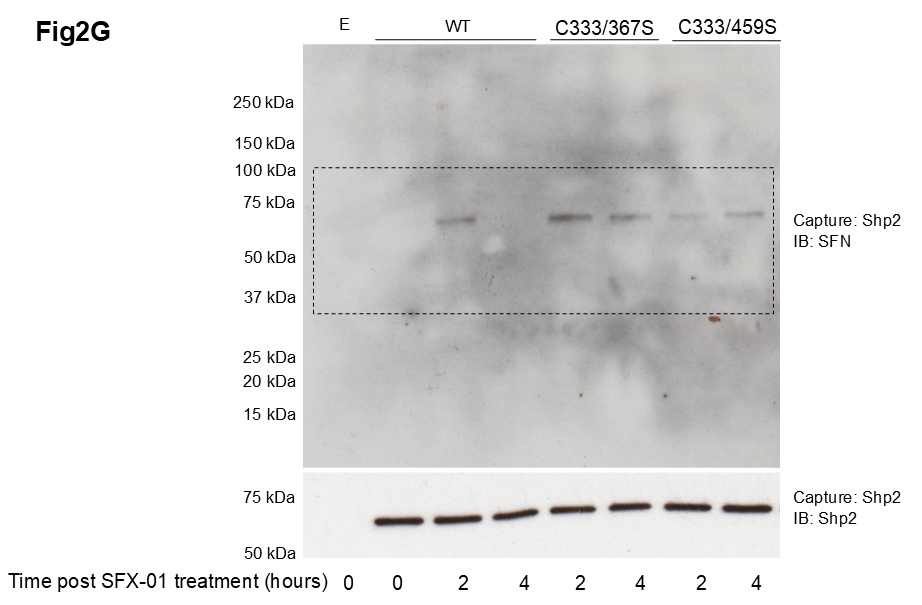

Supplement: Supplementary file 5 — Source data Fig. 2 [file 44321_2025_267_MOESM5_ESM.zip › Fig2/Fig2G.tif]

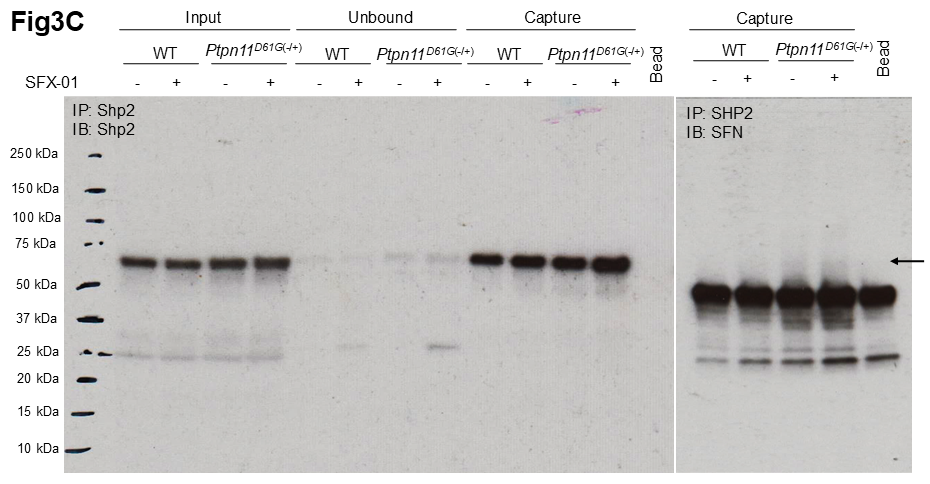

Supplement: Supplementary file 6 — Source data Fig. 3 [file 44321_2025_267_MOESM6_ESM.zip › Fig3/Fig3C.tif]

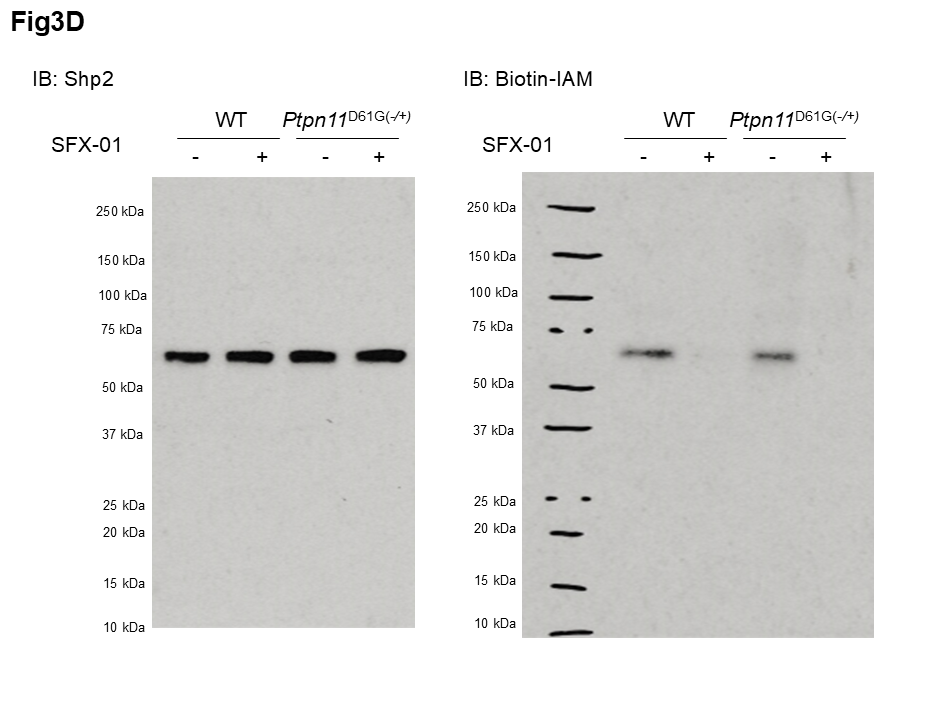

Supplement: Supplementary file 6 — Source data Fig. 3 [file 44321_2025_267_MOESM6_ESM.zip › Fig3/Fig3D.tif]

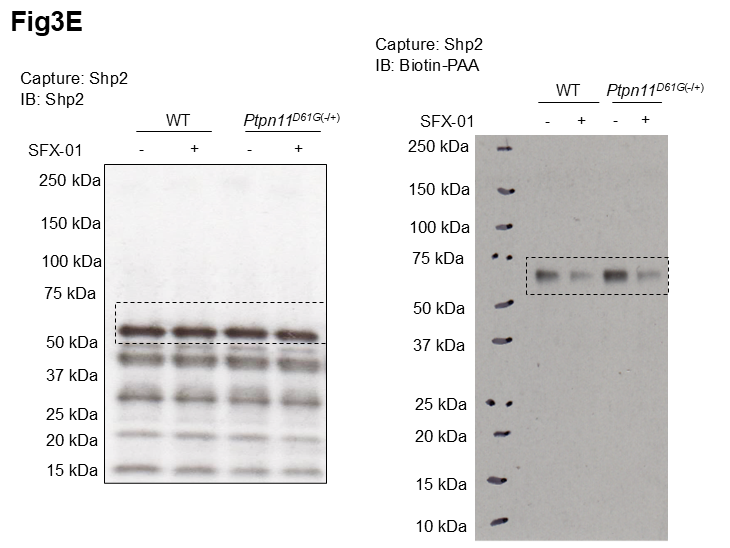

Supplement: Supplementary file 6 — Source data Fig. 3 [file 44321_2025_267_MOESM6_ESM.zip › Fig3/Fig3E.tif]

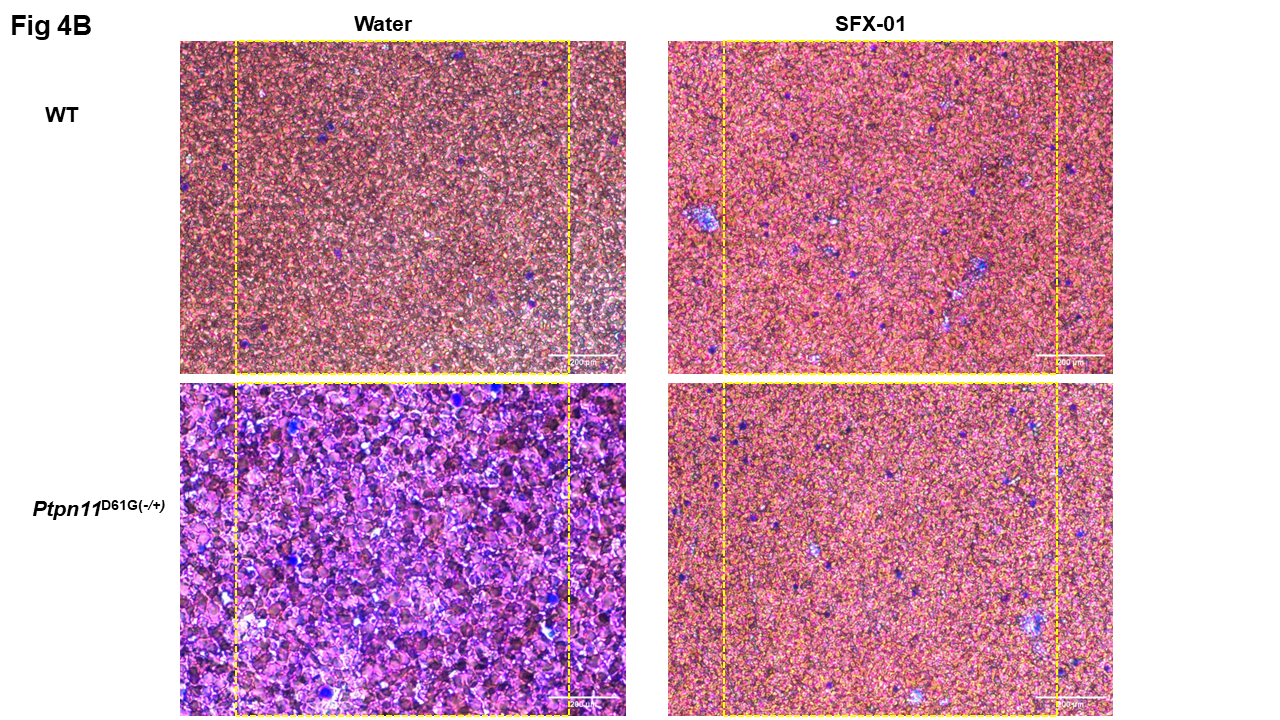

Supplement: Supplementary file 7 — Source data Fig. 4 [file 44321_2025_267_MOESM7_ESM.zip › Fig4/Fig4B.tif]

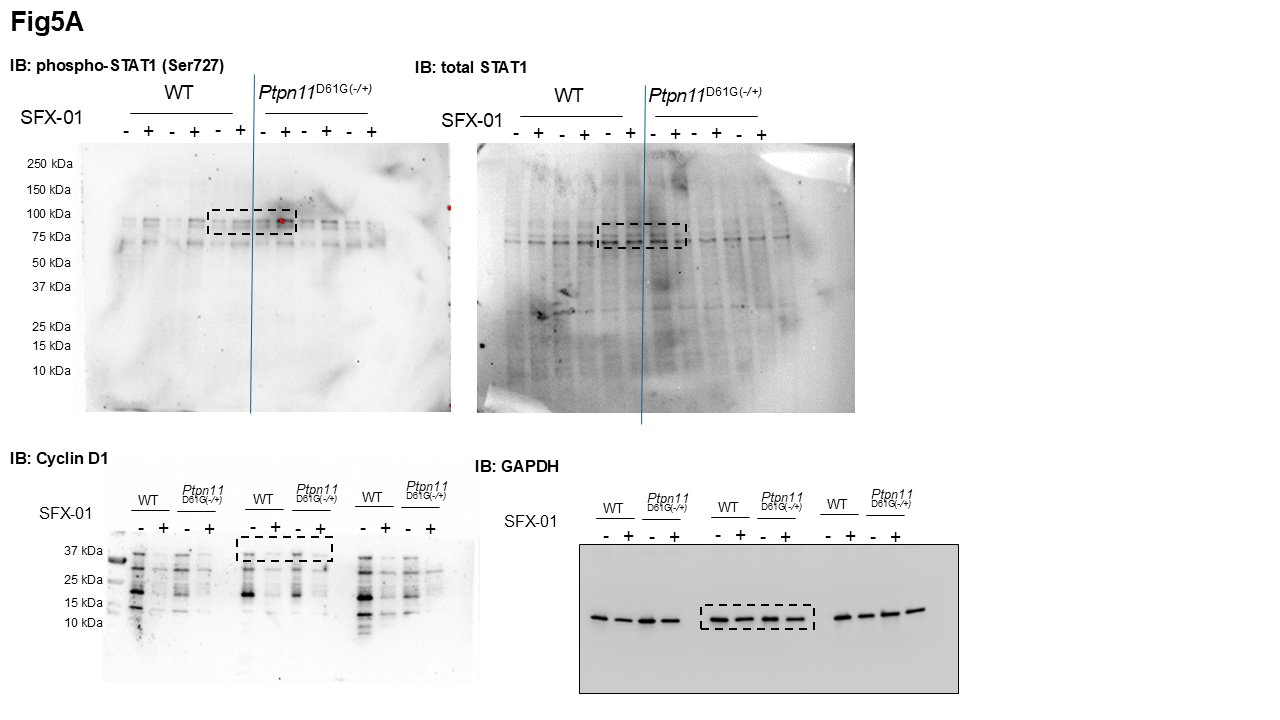

Supplement: Supplementary file 8 — Source data Fig. 5 [file 44321_2025_267_MOESM8_ESM.zip › Fig5/Fig5A.tif]

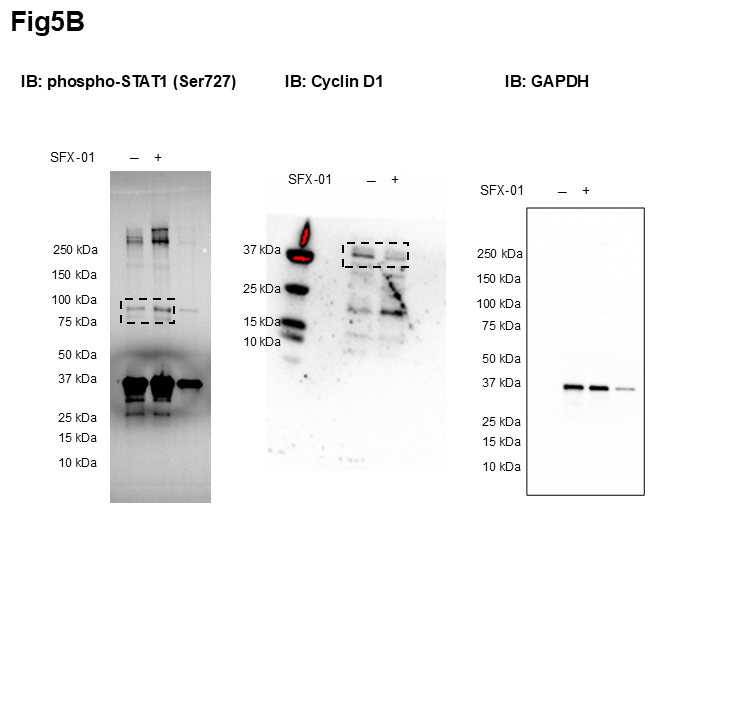

Supplement: Supplementary file 8 — Source data Fig. 5 [file 44321_2025_267_MOESM8_ESM.zip › Fig5/Fig5B.tif]
