# Supplementary material for: SFX-01 is therapeutic against myeloproliferative disorders caused by activating mutations in Shp2
Source: EMBO Mol Med. 2025 Jul 10;17(8):2115–36. doi: 10.1038/s44321-025-00267-7 (PMC12340136; doi:10.1038/s44321-025-00267-7)
Supplement: Supplementary file 9 — Expanded View Figures [file 44321_2025_267_MOESM9_ESM.pdf]

Expanded View Figures

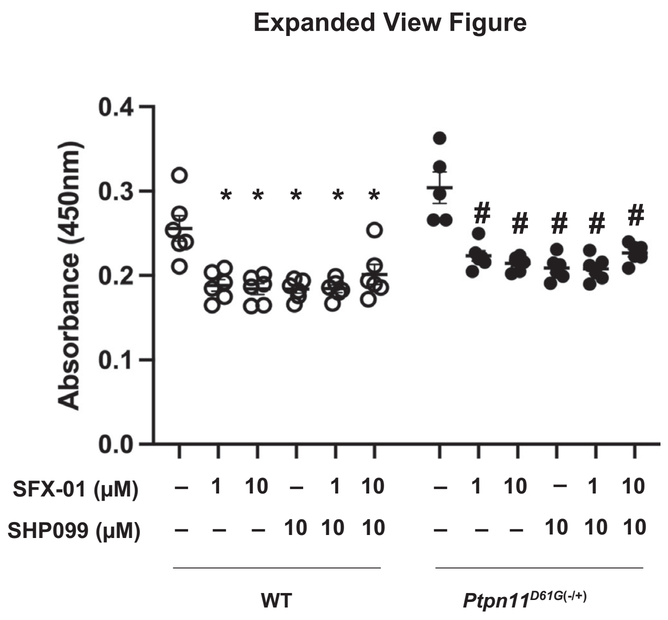

Figure EV1. Combined treatment with SFX-01 and Shp2 did not accentuate their individual attenuation of proliferation.

CD11b+ bone marrow cells isolated from WT and *Ptpn11*$^{D61G(-/+)}$ mice treated with SFX-01, SHP099 or both together. SFX-01 or SHP099 attenuated cell proliferation and this was not accentuated when these interventions were combined. Data are presented as means (± SEM; $n = 5$–6) with P values calculated by two-way ANOVA with Tukey's multiple comparison test. *$P < 0.00001$ versus untreated WT or $^{\#}P < 0.00001$ versus untreated *Ptpn11*$^{D61G(-/+)}$.

 © The Author(s)
